# Electronic Video Consent to Power Precision Health Research: A Pilot Cohort Study

**DOI:** 10.2196/29123

**Published:** 2021-09-08

**Authors:** Arash Naeim, Sarah Dry, David Elashoff, Zhuoer Xie, Antonia Petruse, Clara Magyar, Liliana Johansen, Gabriela Werre, Clara Lajonchere, Neil Wenger

**Affiliations:** 1 UCLA Center for SMART Health Clinical and Translational Science Institute David Geffen School of Medicine at UCLA Los Angeles, CA United States; 2 David Geffen School of Medicine at UCLA Los Angeles, CA United States; 3 Mayo Clinic Rochester, MN United States; 4 Embedded Clinical Research and Innovation Unit CTSI Office of Clinical Research Los Angeles, CA United States; 5 Institute for Precision Health David Geffen School of Medicine at UCLA Los Angeles, CA United States

**Keywords:** biobanking, precision medicine, electronic consent, privacy, pilot study, video, consent, precision, innovation, efficient, precision medicine, cancer, education, barrier, engagement, participation

## Abstract

**Background:**

Developing innovative, efficient, and institutionally scalable biospecimen consent for remnant tissue that meets the National Institutes of Health consent guidelines for genomic and molecular analysis is essential for precision medicine efforts in cancer.

**Objective:**

This study aims to pilot-test an electronic video consent that individuals could complete largely on their own.

**Methods:**

The University of California, Los Angeles developed a video consenting approach designed to be comprehensive yet fast (around 5 minutes) for providing universal consent for remnant biospecimen collection for research. The approach was piloted in 175 patients who were coming in for routine services in laboratory medicine, radiology, oncology, and hospital admissions. The pilot yielded 164 completed postconsent surveys. The pilot assessed the usefulness, ease, and trustworthiness of the video consent. In addition, we explored drivers for opting in or opting out.

**Results:**

The pilot demonstrated that the electronic video consent was well received by patients, with high scores for usefulness, ease, and trustworthiness even among patients that opted out of participation. The revised more animated video pilot test in phase 2 was better received in terms of ease of use (*P*=.005) and the ability to understand the information (*P*<.001). There were significant differences between those who opted in and opted out in their beliefs concerning the usefulness of tissue, trusting researchers, the importance of contributing to science, and privacy risk (*P*<.001). The results showed that “I trust researchers to use leftover biological specimens to promote the public’s health” and “Sharing a biological sample for research is safe because of the privacy protections in place” discriminated opt-in statuses were the strongest predictors (both areas under the curve were 0.88). Privacy concerns seemed universal in individuals who opted out.

**Conclusions:**

Efforts to better educate the community may be needed to help overcome some of the barriers in engaging individuals to participate in precision health initiatives.

## Introduction

Informed consent for biospecimens is an essential component for a robust program in precision medicine (PM). The use of deidentified remnant (leftover) biospecimens has come under recent scrutiny. Although the Notice of Proposed Rule Making (NPRM) to Human Subject Federal Regulations (common rule) [1] considers such tissue as not “human subjects” research, the National Institutes of Health (NIH) Genomic Data Sharing Policy expects informed consent for future research use and broad data sharing to be obtained even if the cell lines or clinical specimens are deidentified [2] (see Textbox 1 for a summary of key components of a broad consent for biospecimens). Moreover, there are many advocates and ethicists who feel there is an obligation to communicate that tissue may be used for research and to obtain informed consent [3]. Patients also want the opportunity to have their preferences dictate the use of clinical specimens for research [4].

**Elements of board consent.**

**General requirements of study-specific informed consent**

1. Obtaining the legally effective informed consent of the participant or the participant’s legally authorized representative

2. Seeking informed consent under circumstances that provide an opportunity to discuss and consider whether or not to participate and that minimize the possibility of coercion or undue influence

3. Providing information in understandable language

4. Providing information that a reasonable person would want to have to make an informed decision about whether to participate and providing an opportunity to discuss that information

5. Avoiding exculpatory language: Exculpatory language either waives or appears to waive the participant’s legal rights or it releases or appears to release the investigator, the sponsor, the institution, or its agents from liability for negligence.

**Basic elements of study-specific informed consent**

6. A description of any reasonably foreseeable risks or discomforts to the participant

7. A description of any benefits to the participant or to others that may reasonably be expected from the research

8. A statement describing the extent, if any, to which confidentiality of records identifying the participant will be maintained

9. A statement that participation is voluntary and that the participant may choose not to participate or discontinue participation at any time without penalty or loss of benefits to which the participant is otherwise entitled

10. A statement that the participant's biospecimens—even if identifiers are removed—may be used for commercial profit

11. A statement about whether the participant will or will not share in the profit

12. A statement indicating if the research will or might include whole genome sequencing

**Unique elements of a broad universal consent for biospecimens**

13. A statement describing the types of research that may be conducted, and the information must be sufficient for a reasonable person to conclude that he or she would consent to the types of research anticipated

14. A statement describing if possible future research could raise particularly sensitive ethical, moral, religious, or cultural issues, in addition to a statement that advises the participant of the possibility that he or she might have chosen not to consent to some of those specific research studies that will use the biospecimens

15. A statement describing the identifiable private information or identifiable biospecimens that might be used in research, whether sharing of the information or biospecimens might occur, and the types of institutions or researchers that might conduct research with the information or biospecimens

16. A statement describing how long the information or biospecimens may be stored and maintained and how long the information or biospecimens may be used for research purposes; these time periods may be indefinite

17. A statement that clinically relevant research results may not be disclosed to the participant

18. A statement informing the participant whom to contact for answers to questions about the participant’s rights regarding storage and use of information or biospecimens and whom to contact regarding research-related harm

Traditionally, in-person paper consents are often resource intensive, not easily scalable, and preclude digital responses from being incorporated in the electronic health record and laboratory information management systems. Given that PM requires large-scale patient engagement, innovations in consenting in conjunction with broad public education [5,6] are required. The emergence of digital health plays a substantial role in defining population-based approaches to electronic consent. Interactive and multimedia slideshow consents have been used for enrollment of participants in biobanks [7,8], but such slideshow consents require increased participant time. Animated video consent approaches have been effective in providing comprehensive information and improving participants’ understanding of content [9,10], but such video consents have not been used in biobank research associated with PM.

EngageUC, an NIH-funded study, examined biobanking in the University of California system with community constituents to better define the innovative and accessible consent materials needed as part of a scalable institutional biobanking program in support of PM [4,11]. The following key themes emerged: the public should be educated about biobanking, consent content source should be considered knowledgeable and trustworthy, consent process should be low stress with an opportunity to get answers to questions, format and language of the consenting material should be easy to understand, and oversight should be conducted by the community and stakeholders.

In this study, we engaged the community and stakeholders across the University of California, Los Angeles (UCLA) Health System, David Geffen School of Medicine at UCLA, UCLA Institute of Precision Health, and the UCLA Clinical and Translational Science Institute (CTSI) to create and pilot an innovative potentially scalable universal video consent that asks patients to give a “broad” or “one time” consent that allows researchers to use their biomaterial and clinical data in a manner that meets the criteria defined by both the NIH and NPRM [1].

## Methods

This study was approved by the UCLA Institutional Review Board (IRB; #15-001395IRB) with a waiver of written informed consent.

### Governance Structure

We formulated a strong governance structure, including a community advisory board (CAB), to oversee and give feedback on the consent design and process [11].

#### Community Advisory Board

Our study team assembled a CAB consisting of 11 respected leaders that were highly involved with organizations in the Los Angeles region that understood our diverse communities and represented their perspectives. The members were racially diverse (2 were African American, 2 were Latinx, 1 was Asian American, 1 was Native American, 1 was Persian-American, and 4 were non-Hispanic White) and equitable with respect to gender (5 were males and 6 were females).

The committee held five meetings between July 2015 and June 2019 to review and guide the video design. The CAB’s primary focus was to ensure the video was easy to understand and explained the purpose of the consent. The board additionally focused on three key areas: inclusion of diverse patients in PM education, outreach, and research; integration of research and clinical operations; and potential return of genetic results.

#### Internal Advisory Board

The internal advisory board included our institutional research leaders from the David Geffen School of Medicine, Institute of Precision Health, CTSI, UCLA Health, and additional faculty with expertise in bioethics, patient engagement, biobanking, and IRB. The members of the internal advisory board provided substantial feedback to ensure the video content was informative, met NIH standards, addressed both genetic testing and the potential for collaborations with external companies and federal partners, was culturally sensitive, and represented the diversity of Los Angeles.

### Video Development

The content for both the text and animated video consents were adapted from paper versions of a biobanking consent developed by EngageUC [4]. The animated consent included a statement about collaboration with governmental agencies, commercial entities, and other academic institutions, and a statement that potential secondary use of data could include genomic sequencing. The videos were targeting a seventh grade reading level. Both video consents were designed to be 4 to 5 minutes in length. These pilot videos were in English and Spanish, with voiceovers for the animated consent. All the essential components for an NIH informed consent were included in the videos (see Textbox 1) [12].

#### Phase 1

**Figure 1:**
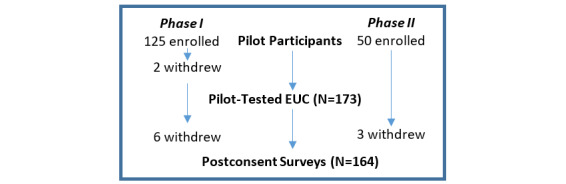
Pilot-testing electronic universal consent (EUC).

A text-based video (text moving from screen to screen) was first designed to consent patients around the use of their remnant tissue for research. A convenience sample of 125 patients were enrolled but only 123 completed postconsent surveys (see Figure 1).

#### Phase 2

**Figure 2:**
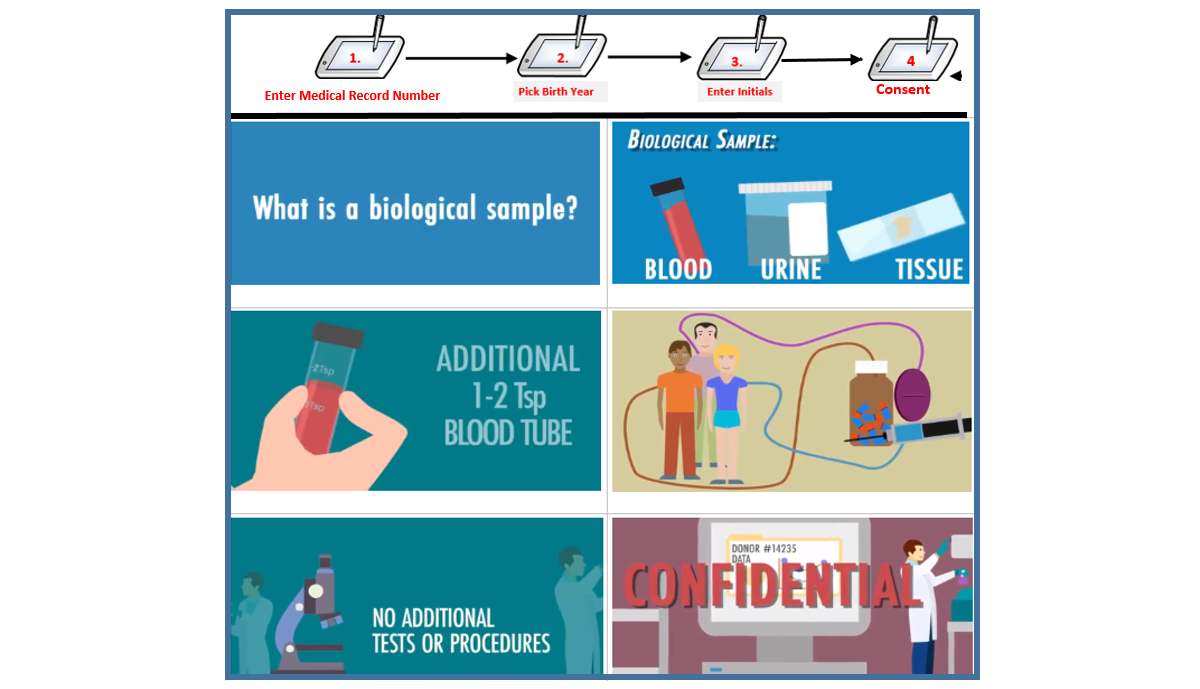
Electronic universal consent flow and representative screenshots.

Our CAB and internal advisory board guided the adaptation of the universal consent video to a fully animated (cartoon-like) video to better communicate content to lay audiences and use this to power the Institute of Precision Health ATLAS biobank (see Figure 2). The video conveyed that this sample would be collected at one time and as a piggyback to any standard lab draw or intravenous placement. For this phase, an additional convenience sample of 50 patients were enrolled, of whom 47 completed postconsent surveys (see Figure 1). Phase II pilot testing was mainly to evaluate if the additional animation improved the user experience of the consent video.

### Consenting Process

The first pilot was conducted at five distinct locations within UCLA Ronald Reagan Hospital: (1) hospital admissions, (2) clinical lab, (3) mammography clinic, (4) oncology clinic, and (5) liver clinic. Technical assistance was available at all these locations. Sites were selected because of their diverse populations and high volume (eg, mammography). All patients were approached and technical assistance from study staff was made available. The second pilot was expanded to include perioperative suites. Patients had to validate their identity before viewing the video and responding to the consent questions (see Figure 2).

Patients were asked to choose their preferred language (English or Spanish) and then validate their identity by entering their medical record number, selecting their birth year (out of 6), and entering the initials of their first and last name. Once validated, individuals could view the video and provide consent. There was no prompting from study staff or clinic personnel. A paper brochure with frequently asked questions (FAQs; available in English and Spanish) was handed to patients with the iPad. Both the video and the FAQs let patients know they could change their consent status at any time. After watching the video, patients were asked: where they wanted to opt in or opt out of having remnant biospecimens used for research and if they would be open to recontact for future research.

### Data Collection and Outcome Measures

Demographic data, including age, race or ethnicity, highest level of education, and language preference for the convenience sample, were collected.

A postconsent survey was developed in English and Spanish to evaluate the effectiveness of the universal consent videos and understand drivers of consent choice. Patients were approached after completing the universal consent videos. Volunteers received a US $5 dollar Target gift card to compensate them for their time. *Patient impressions* of the universal consent videos were evaluated with three questions (how useful did you find the information, how easy was the information to understand, and how much did you feel you could trust the information) using a five-point Likert scale (not at all, not really, somewhat, mostly, and very).

#### Drivers of Consent Choice

The internal advisory board helped develop questions used to understand the reasons patients’ opted in or out. Individuals who *opted in received additional questions* to determine drivers (hoping the research will help me in the future, hoping the research will help my family and friends in the future, hoping to advance science, or hoping to find a cure for a disease). Individuals who *opted out received additional questions* to determine drivers (do not want my tissue used for anyone else, concerns about privacy, concerns that a product will be made with my tissue and I will not benefit, or did not understand what I was being asked).

#### Patient Health Beliefs Regarding Medical Research

The study team developed a 10-item questionnaire to evaluate patients’ beliefs about biomedical research using validated instruments as guides [13-16]. This new survey measured attitudes about science, optimism, altruism, privacy, social support, justice, and conflict of interest.

### Opt-in or Opt-out Status

The patient’s decision to either opt in or opt out of sharing their remnant samples was recorded. We also tracked the number of patients who agreed to be contacted for future research.

### Statistical Analyses

Demographic information, consent rates, and patient ratings about consent were summarized using descriptive statistics including medians, ranges, and percentages. The video script was run through the Flesh-Kinkaid Readability Test tool in Word (Microsoft Corporation) to determine the grade level of the universal consent videos. The patient characteristics between those that opted in and opted out were compared with chi-square tests for homogeneity. The consent rates were compared between phases 1 and 2 consents with chi-square tests for homogeneity. Patients’ evaluation of the usefulness, ease, and trustworthiness of the videos in phases 1 and 2 were compared using Wilcoxon rank sum tests since the variables had skewed distributions. To determine the internal consistency of the 10-item beliefs survey, Cronbach alpha was used. Univariate logistic regression was used to examine the association between patients’ beliefs and their consent decision. We used the AUC receiver operating characteristic curves to predict which patients opted in versus opted out. Two-sided *P* values were reported, and variables were considered statistically significant if the *P* value was <.05. All analyses were conducted using Stata 15 (StataCorp) [17].

## Results

### Community Advisory Board Suggestions

The CAB played a significant role in the video design, which made the video rich in content, ensured that the language was appropriate for the lay population, addressed the most concerning questions from the community, was applicable to a diverse population, and was less than 5 minutes in duration. The board’s primary focus was to ensure that the video was understandable and appropriately explained why UCLA was asking them to donate biosamples and clinical data for research. The board additionally focused on three key areas: (1) how to ensure the inclusion of diverse patients and communities in PM program education, outreach, and research; (2) if and how to return PM research findings to individual patients who contribute samples and data to the biobank; and (3) how to appropriately bridge research and clinical operations.

### Participants

A total of 175 patients enrolled across the two pilot phases, of which 173 actually went through the electronic video consent (see Figure 1). The population was mostly middle age (n=130, 75% were younger than 60 years), female (n=123, 69%), White (n=86, 50%), and educated (n=104, 60% had at least college education; Table 1). The majority of patients preferred English (n=161, 93%). There were no significant differences for age, education, gender, or race between patients who opted in or opted out.

**Table 1:** Sociodemographic data (cohort that tested consent).

Demographic		Participants (N=173), n (%)
**Age (years)**		
	<30	36 (20.8)
	30-39	37 (21.4)
	40-49	25 (14.5)
	50-59	33 (19.1)
	60-69	20 (11.6)
	70-79	20 (11.6)
	≥80	2 (1.2)
**Gender**		
	Male	50 (30.6)
	Female	123 (69.4)
**Race/ethnicity**		
	White	86 (49.7)
	Asian	29 (16.9)
	Black	21 (12.2)
	Hispanic	31 (18.0)
	Native American	1 (0.6)
	Other	5 (2.3)
**Education**		
	Less than high school	8 (4.7)
	High school graduate	30 (17.7)
	Some college	28 (16.5)
	College graduate	53 (31.2)
	Master’s degree	27 (15.9)
	MD or PhD	24 (14.1)
	Unknown	3 (1.7)
**Language**		
	English preferred	161 (93.1)
	Spanish preferred	12 (6.9)

### Consent Rate

There was no significant difference for consent rate between the two phases (44/50, 88.0% vs 112/ 123, 91.1%; *P*=.41). Across the entire cohort, 56% (97/173) of individuals agreed to be recontacted to participate in other biomedical research projects.

### Patients’ Health Beliefs on PM Research

The 10-item questionnaire had good internal consistency with an alpha coefficient of .93, which means the results were consistent among similar questions. Univariate logistic regression analysis showed that there were significant differences on all 10 items between the groups who opted in versus opted out (all *P*<.001). Additionally, we calculated AUC to evaluate the ability of the questions to discriminate which question predicted patients opting in. The results showed that “I trust researchers to use leftover biological specimens to promote the public's health” and “Sharing a biological sample for research is safe because of the privacy protections in place” discriminated opt-in statuses were the strongest predictors (both AUC were 0.88; Table 2).

**Table 2:** The association between participants’ health beliefs and demographic characteristics with their decision to opt in (N=164).

Health beliefs and demographics		Construct	Odds ratio (95% CI)	AUC^a^
Q1. Results of research using biological samples will help future generations.		Attitude toward science, optimism, altruism	5.5 (2.6-12.2)*	0.8*
Q2. It is important for individuals to participate in research to advance science.		Altruism, communitarianism	10 (4.1-24.5)*	0.87*
Q3. Sharing a biological sample for research is safe because of the privacy protections in place.		Attitude toward science, privacy concerns	4.5 (2.4-7.5)*	0.88*
Q4. Results of the research using donated biological samples will help me or my family in the future.		Attitude toward science, optimism, altruism	4.5 (2.3-8.3)*	0.84*
Q5. My family and friends support donating biological samples for research.		Social support	3 (1.7-5.4)*	0.8*
Q6. Research on donated tissue may lead to medical breakthroughs from which UCLA^b^ and researchers will profit.		Attitude toward science, justice, conflict of interest	3 (1.7-5.1)*	0.76*
Q7. I trust researchers to use leftover biological specimens to promote the public’s health.		Attitude toward science, justice, trust	5.5 (2.7-11)*	0.88*
Q8. The most important thing to researchers is helping people and curing disease.		Attitude toward science	5 (2.4-10)*	0.79*
Q9. People have a responsibility to help each other.		Altruism	5.5 (2.5-11)*	0.8*
Q10. If a person does not donate tissue for research it just goes to waste.		Attitude toward science	3.3 (1.9-6.2)*	0.81*
Age		N/A^c^	1.3 (0.9-1.7)	0.62
Education		N/A	0.96 (0.7-1.3)	0.52
**Race**		N/A		0.54
	White		1.00 (reference)	
	Asian		0.56 (0.17-1.84)	
	Black		0.49 (0.13-1.78)	
	Hispanic		1.76 (0.36-8.66)	
	Others		0.36 (0.03-3.89)	
Gender (female)		N/A	0.7 (0.3-1.9)	0.54

^a^AUC: area under the curve.

^b^UCLA: University of California, Los Angeles.

^c^N/A: not applicable.

*P<.001

### Evaluation of the Universal Consent Video

We also examined whether there was a difference between the video consent evaluations of patients who opted in and opted out regarding the ease of use, usefulness, and the trustworthiness as three outcomes: useful and not useful, easy to understand and not easy to understand, and trustworthy and not trustworthy, respectively. In terms of where it was useful or easy to understand, the universal consent video did not differ between two groups (those who opted in vs opted out). However, 88.4% (136/158) of the patients who opted in felt they could trust the information in the video compared to only 53.3% (8/15) of the patients who opted out (*P*<.001).

We compared the evaluations of the phase 1 text-based video and the phase 2 animated video among patients regarding the ease of use, usefulness, and trustworthiness. We found that there was a statistically significant difference between the text-based video and the animated video regarding the ease of use (*P*=.005) and the ability to understand this information (*P*<.001). There was no significant difference regarding the trustworthiness between the text-based video and animated video (*P*=.20; Table 3).

**Table 3:** Comparison of usefulness, ease of use, and trustworthiness between two pilot phases of video consent (N=164).

Variables	Phase 1 (n=117)^a^, median (IQR)	Phase 2 (n=47)^a^, median (IQR)	*P* value
Usefulness	4 (3-5)	5 (4-5)	.005
Ease	5 (4-5)	5 (5-5)	<.001
Trustworthiness	4.5 (4-5)	5 (4-5)	.20

^a^Responses were based on a 5-point Likert scale: 1 (not at all), 2 (not really), 3 (somewhat), 4 (mostly), and 5 (very).

### Important Factors for Opting In and Opting Out

Questions that garnered a large majority of patients (≥80%) responding as “moderate” or “very important” were a key focus. Among the four questions we asked the patients who opted in (Table 4), three of four made this threshold: “research benefiting me,” “hoping PM research could advance science,” and “cure diseases.” Among the four questions we asked the patients who opted out (Table 5), only 1 question about “privacy” made this threshold and was a factor for all the patients.

**Table 4:** Reasons for opting in (n=101 completed).

Reasons for opting in	Not at all, n (%)	A little, n (%)	Moderate important, n (%)	Very important, n (%)
Hoping the research will help me in the future	6 (5.9)	10 (9.9)	27 (26.7)	58 (57.4)
Hoping the research will help family, friends, or others in the future	0 (0)	4 (4)	13 (12.9)	84 (83.1)
Hoping to advance science	0 (0)	2 (1.9)	9 (8.9)	90 (89.1)
Hoping to contribute to the cure of disease^a^	0 (0)	2 (2)	4 (4)	94 (94)

^a^One patient did not answer the question.

**Table 5:** Reasons for opting out (n=20 completed).

Reasons for opting out	Not at all, n (%)	A little, n (%)	Moderate important, n (%)	Very important, n (%)
Do not want my tissue used for anyone else^a^	6 (31.6)	2 (10.5)	6 (31.6)	5 (26.3)
Concern about privacy	0 (0)	0 (0)	2 (10)	18 (90)
Concern that a product may be made from my tissue and I will not benefit^a^	5 (26.3)	4 (21.1)	2 (10.5)	8 (42.1)
Did not understand what I was asked to consent to^b^	8 (44.4)	4 (22.2)	4 (22.2)	2 (11.1)

^a^One patient did not answer the question.

^b^Two patients did not answer the question.

## Discussion

Our study indicated that our universal consent animated video is easy and informative. Because it is short and self-administered, this is a possible solution for a scalable consent method for population-based PM research. Compared to in-person paper consent, electronic video consent requires fewer human resources and less physical space. As designed in this study, it could be deployed to any number of devices and applied at multiple medical locations. Hence, it is suitable for large-scale efforts to collect informed consent from a large population with a modest incremental cost. Furthermore, it allows patients a safe space to participate in the consenting process without the pressure an in-person process might create. To apply it broadly and effectively to diverse populations, it is critical that the universal consent video addresses potential concerns participants may have about the research project to build trust, reassure potential participants about privacy concerns, be transparent (which further increases trust), and address the potential of the research.

In line with other studies, we found that trust is one of the most important factors for patients opting in to biomedical research [18]. Multiple studies have identified reasons for reduced trust between patients and researchers: participants are not clear about their rights over their data in the biobank [19]; patients did not understand biobanking or the aims of the clinical trial [20]; patients might have concerns about allowing researchers to use their data for the unforeseen secondary research via a broad consent process [21]; patients who consented to participate in clinical trials heavily depended on how much they trusted the physician [22], whereas in this consent process, there are no health professionals communicating with patients; or there is no immediate benefit for patients in PM research.

Delivering comprehensive information about biobanking and PM research is necessary for truly informed consent and to build patients’ trust. However, it is important to balance the video content and length, as patients might lose interest or read or watch the consent cursorily if it is too long or if the content is not presented in language that average individuals can understand [23-25]. One solution to increase patients’ understanding of and trust in PM research and biobanking may be to provide more concrete examples of clinical research and PM. A complementary approach may be to provide personal stories of successful PM in UCLA patients. Such educational videos could help interested individuals learn more about the value of remnant biospecimens, clinical data, and clinical research in advancing science. It is important to ensure patients understand that PM research takes time, so the benefits of participation will not be immediate.

In this study, all (100%) patients who opted out responded that concerns about privacy were moderately or very important to them. This is consistent with results from multiple studies suggesting that patients were concerned about misuse of their personal data [26]. If patients do not understand how their data might be used or who might use the data, they are less likely to give permission to share the data [27]. As PM research requires hundreds and thousands (or more, depending on the specific question) of unique biological samples, emphasizing how clinical information will be protected should be embedded in the consent process. Furthermore, a transparent policy to efficiently manage data access and protect individual’s privacy through a variety of data access controls and an oversight committee for ethical governance of the biobank is a necessity [4]. In fact, some authors believe this represents the only way to build public trust and protect participants’ privacy [28]. Researchers, scientists, and policy makers should embrace the notion that that if privacy concerns are well addressed in the consent and clearly communicated in a trustworthy way, this could enhance potential participants’ understanding of and trust in the research process.

Our study found that potential participants’ health beliefs were the most significant driver of their willingness to participate in a precision health initiative. Patients who opted in believed that their participation could advance science, find cures for disease, and help others. This confirms previous studies that participation in biobank research was based on altruistic motivations and responsibilities to assist future generations [18]. Together with early studies, our findings suggested that emphasizing the importance of patients’ participation to benefit others and contribute to science is associated with the high participation rate in clinical research. From these 10 health belief questions, we again confirmed that if patients trust the researchers and believe their personal privacy is protected, they are more likely to donate their biospecimens.

This pilot study has its limitations. This study only included a convenience sample of patients who agreed to do the electronic consent and answer the additional survey. The sample size was small, and there were smaller subgroups in each category of race, age, gender, and educational level, which limited our ability to evaluate any differences between these populations. We also did not evaluate the participants’ health statuses, which prevented us from understanding if differences in consent rate and health beliefs exist among patients with different diagnoses or disease burden. Future research needs to evaluate the electronic video consent performance in a larger population, so these and other potentially important variables such as low health literacy can be studied more comprehensively.

In summary, we created and piloted an innovative electronic video consent that was self-administered and easy to understand for patients. This approach will next be tested for scalability as an enterprise solution by expanding across 18 clinical sites across the UCLA health system. Future goals include expansion to other University of California sites and piloting the video and process in affiliated county hospitals within the larger Los Angeles County. We believe our video consent and process offer an approach that would allow for more robust inclusion of institutions that do not have the financial resources to use employees for in-person consent. Given the reality that many such institutions will serve patients who are chronically ill, of lower socioeconomic status, and who are from underrepresented minority populations, our video consent and process offer the possibility for these groups to become better represented in PM research. The importance of participation in PM remain unclear especially among ethnic minority populations.

## Abbreviations

AUC: area under the curve
CAB: community advisory board
CTSI: Clinical and Translational Science Institute
FAQ: frequently asked question
IRB: Institutional Review Board
NIH: National Institutes of Health
NPRM: Notice of Proposed Rule Making
PM: precision medicine
UCLA: University of California, Los Angeles
