# Correction: Electronic Video Consent to Power Precision Health Research: A Pilot Cohort Study

**DOI:** 10.2196/33891

**Published:** 2021-10-21

**Authors:** Arash Naeim, Sarah Dry, David Elashoff, Zhuoer Xie, Antonia Petruse, Clara Magyar, Liliana Johansen, Gabriela Werre, Clara Lajonchere, Neil Wenger

**Affiliations:** 1 UCLA Center for SMART Health Clinical and Translational Science Institute David Geffen School of Medicine at UCLA Los Angeles, CA United States; 2 David Geffen School of Medicine at UCLA Los Angeles, CA United States; 3 Mayo Clinic Rochester, MN United States; 4 Embedded Clinical Research and Innovation Unit CTSI Office of Clinical Research Los Angeles, CA United States; 5 Institute for Precision Health David Geffen School of Medicine at UCLA Los Angeles, CA United States

In “Electronic Video Consent to Power Precision Health Research: A Pilot Cohort Study” (JMIR Form Res 2021;5(9):e29123) the authors noted six errors.

The title of the originally published article appeared as follows:

Electronic Video Consent to Power Precision Research: A Pilot Cohort Study

In the corrected version, the title has been revised to:

Electronic Video Consent to Power Precision Health Research: A Pilot Cohort Study

In the original version, author Liliana Johansen's name was displayed incorrectly as follows:

Lilliana Johansen

In the revised version, it has been corrected as follows:

Liliana Johansen

In the originally published paper, Affiliation 1 appeared as follows:

UCLA Center for SMART Health, Clinical Translational Science Institute, David Geffen School of Medicine at UCLA, Los Angeles, CA, United States

In the revised version, it has been revised to:

UCLA Center for SMART Health, Clinical and Translational Science Institute, David Geffen School of Medicine at UCLA, Los Angeles, CA, United States

In the originally published paper, Affiliation 2 appeared as follows:

David Geffen UCLA School of Medicine, Los Angeles, CA, United States

In the revised version, it has been revised to:

David Geffen School of Medicine at UCLA, Los Angeles, CA, United States

In the originally published paper, Affiliation 4 appeared as follows:

Office of Clinical Research, Clinical and Translational Science Institute, David Geffen School of Medicine at UCLA, Los Angeles, CA, United States

In the revised version, it has been revised to:

Embedded Clinical Research and Innovation Unit, CTSI Office of Clinical Research, Los Angeles, CA, United States

In the originally published paper, the following email address of the corresponding address was provided:

arashnaeim@gmail.com

In the revised version, it has been changed to:

anaeim@mednet.ucla.edu

The corrections will appear in the online version of the paper on the JMIR Publications website on October 21, 2021, together with the publication of this correction notice. Because this was made after submission to PubMed, PubMed Central, and other full-text repositories, the corrected article has also been resubmitted to those repositories.

